# Supplementary material for: Hydrophobic Modification of Biopolymer Aerogels by Cold Plasma Coating
Source: Polymers (Basel). 2021 Sep 4;13(17):3000. doi: 10.3390/polym13173000 (PMC8434000; doi:10.3390/polym13173000)
Supplement: Supplementary file 1 [file polymers-13-03000-s001.zip › Supporting_Information_for_Publication_Fromat_MDPI.pdf]

Hydrophobic Modification of Biopolymer Aerogels by Cold Plasma Coating

Baldur Schroeter [1,*], Isabella Jung [1], Katharina Bauer [2], Pavel Gurikov [3] and Irina Smirnova [1]

[1] Institute for Thermal Separation Processes, Hamburg University of Technology, Eißendorfer Straße 38, 21073 Hamburg, Germany; Isabella.Jung@tuhh.de (I.J.); Irina.Smirnova@tuhh.de (I.S.)
[2] Diener Electronic GmbH & Co. KG, Nagolder Straße 61, 72224 Ebhausen, Germany; Katharina-bauer@mein.gmx
[3] Laboratory for Development and Modelling of Novel Nanoporous Materials, Hamburg University of Technology, Eißendorfer Straße 38, 21073 Hamburg, Germany; Pavel.Gurikov@tuhh.de
* Correspondence: baldur.schroeter@tuhh.de

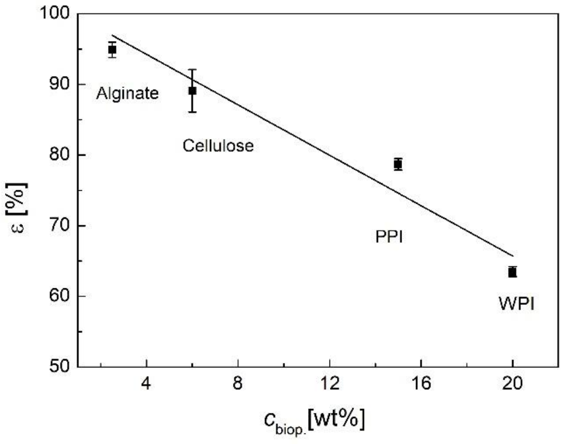

Figure S1. Overall porosity of different aerogels in dependence of the solid content in biopolymer stock solutions. Solid line corresponds to linear fitting, error bars correspond to the density measurement errors.

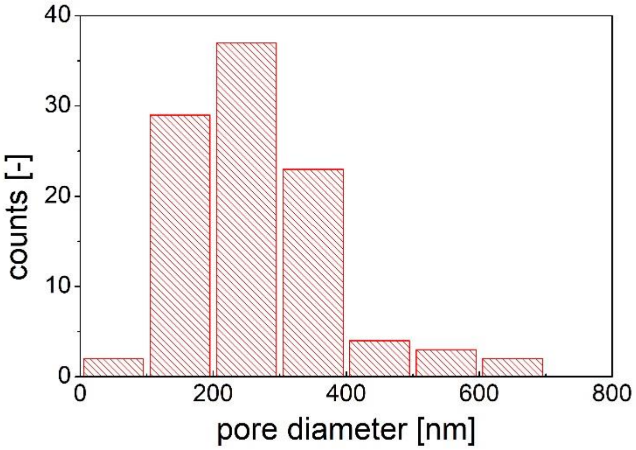

Figure S2. Macropore-distribution in alginate aerogel based on image analysis (100 pores) of SEM pictures.

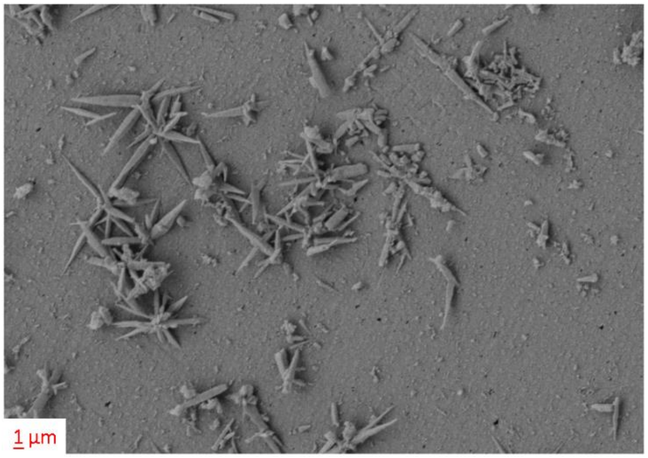

Figure S3. SEM picture of aerogel surface after plasma treatment. WPI aerogel, coating: PFAC-6, mode: CW, p_{input} = 90 W, t = 5 min.

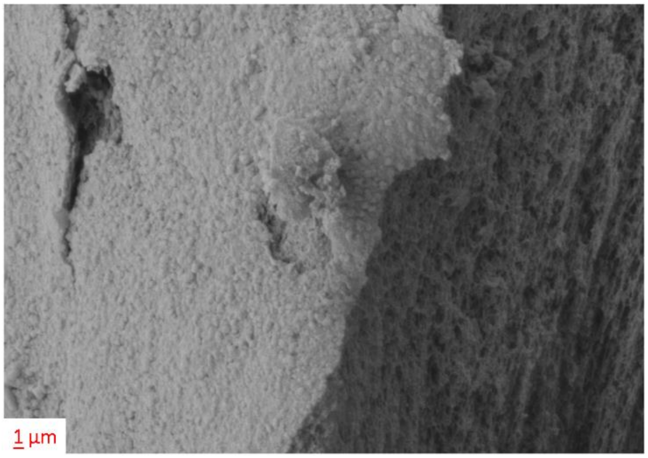

Figure S4. SEM picture of coating layer and aerogel pores after plasma treatment. Alginate aerogel, coating: PFAC-6, mode: CW, p_{input} = 90 W, t = 50 min.

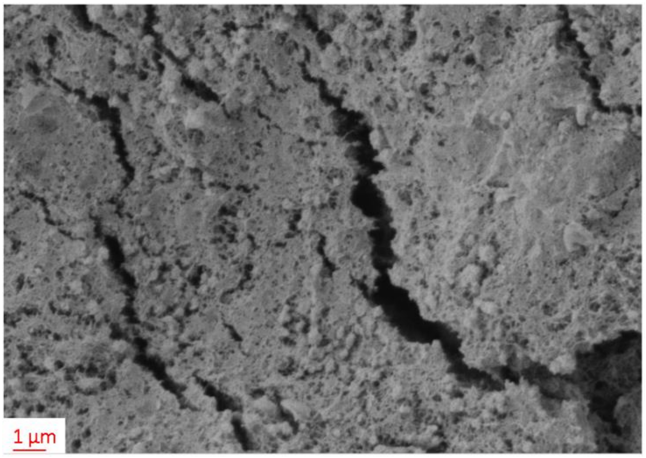

Figure S5. SEM picture of non-enclosed coating layer on aerogel surface after plasma treatment. Cellulose aerogel, coating: PFAC-6, mode: CW, p_{input} = 90 W, t = 5 min.

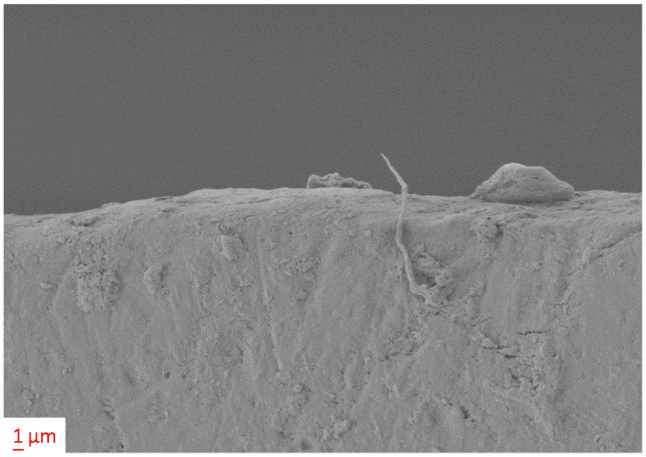

Figure S6. SEM picture of coating layer on aerogel surface after plasma treatment. PPI aerogel, coating: PFAC-6, mode: CW, p_{input} = 90 W, t = 5 min.

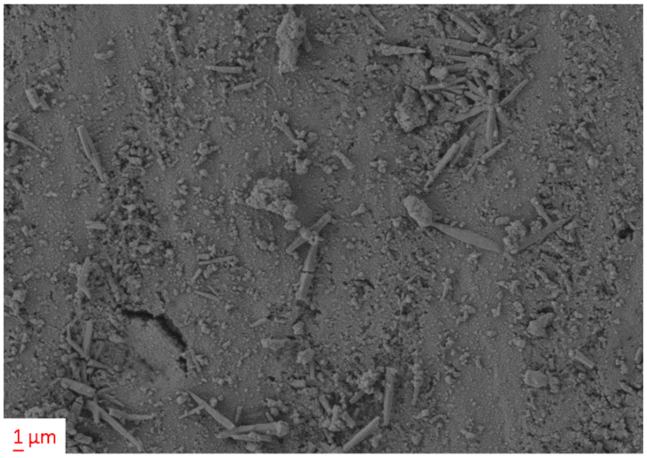

Figure S7. SEM picture of coating layer on aerogel surface after plasma treatment. PPI aerogel, coating: PFAC-6, mode: CW, p_{input} = 90 W, t = 50 min.

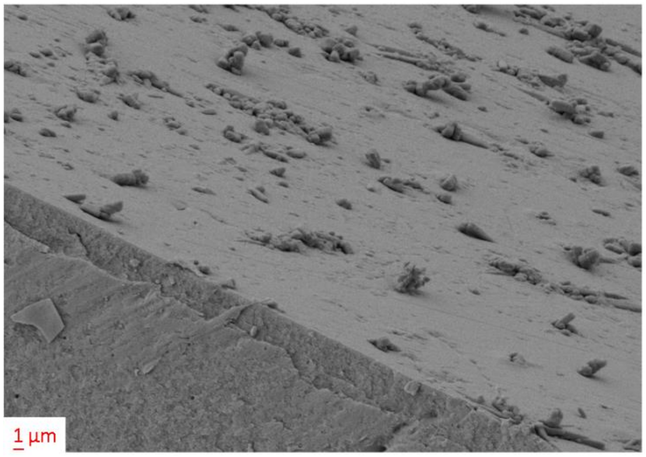

Figure S8. SEM picture of coating layer and aerogel pores after plasma treatment. WPI aerogel, coating: PFAC-6, mode: CW, p_{input} = 90 W, t = 50 min.

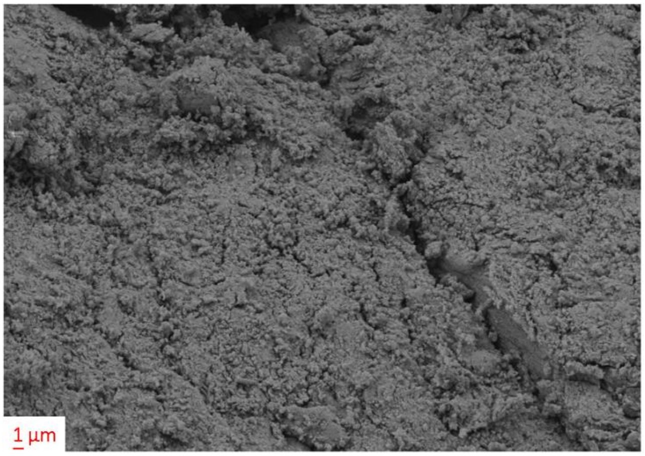

Figure S9. SEM picture of coating layer on aerogel surface after plasma treatment. Alginate aerogel, coating: PFAC-8, mode: CW, p_{input} = 30 W, t = 50 min.

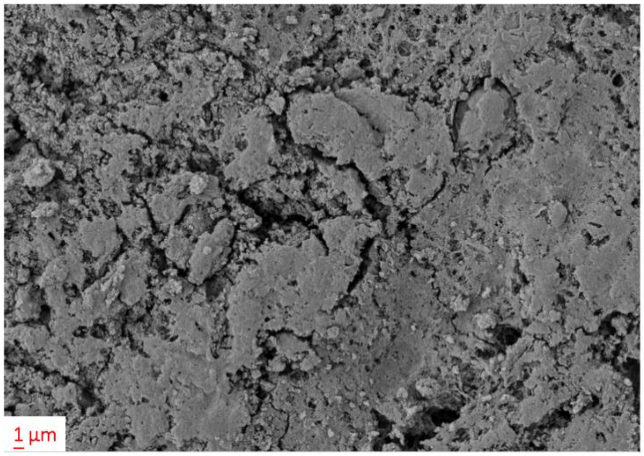

Figure S10. SEM picture of non-enclosed coating layer on aerogel surface after plasma treatment. Cellulose aerogel, coating: PFAC-8, mode: CW, p_{input} = 30 W, t = 50 min.

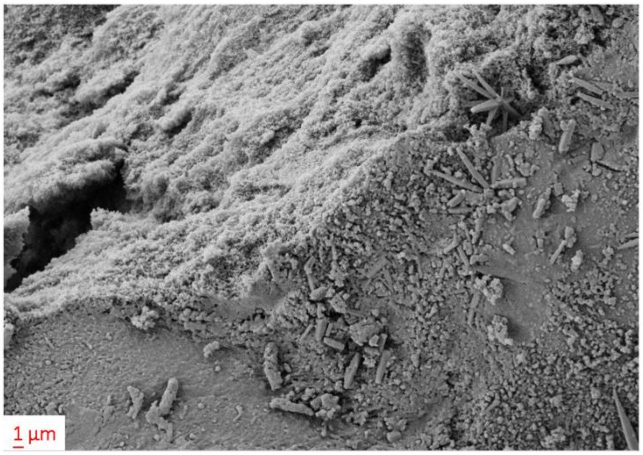

Figure S11. SEM picture of coating layer and aerogel pores after plasma treatment. PPI aerogel, coating: PFAC-8, mode: CW, p_{input} = 30 W, t = 50 min.

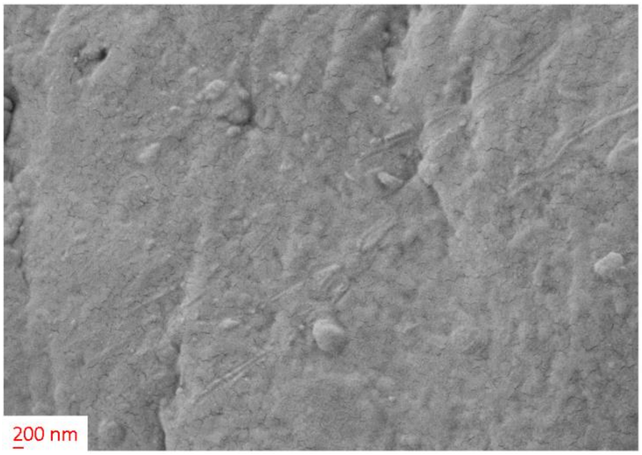

Figure S12. SEM picture of coating layer on aerogel surface after plasma treatment. Alginate aerogel, coating: C_4F_8, mode: CW, p_{input} = 90 W, t = 50 min.

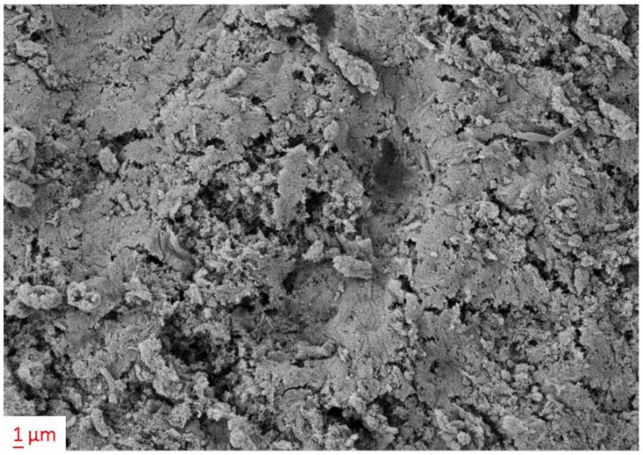

Figure S13. SEM picture of non-enclosed coating layer on aerogel surface after plasma treatment. Cellulose aerogel, coating: C_4F_8, mode: CW, p_{input} = 90 W, t = 5 min.

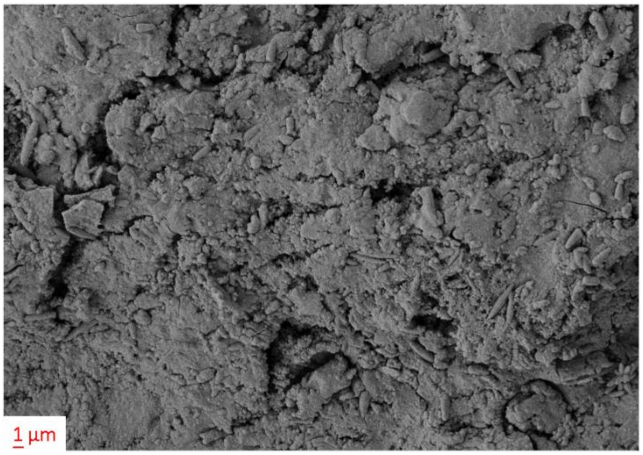

Figure S14. SEM picture of coating layer on aerogel surface after plasma treatment. Cellulose aerogel, coating: C_4F_8, mode: CW, p_{input} = 90 W, t = 50 min.

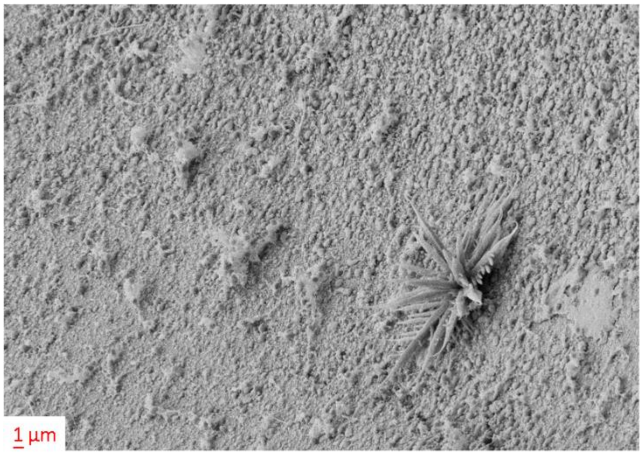

Figure S15. SEM picture of coating and open pores on aerogel surface after plasma treatment. PPI aerogel, coating: C_4F_8, mode: CW, p_{input} = 90 W, t = 5 min.

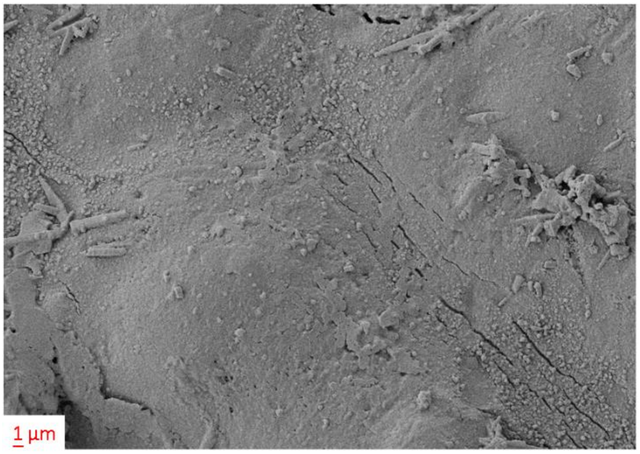

Figure S16. SEM picture of coating layer on aerogel surface after plasma treatment. PPI aerogel, coating: C_4F_8, mode: CW, p_{input} = 90 W, t = 50 min.

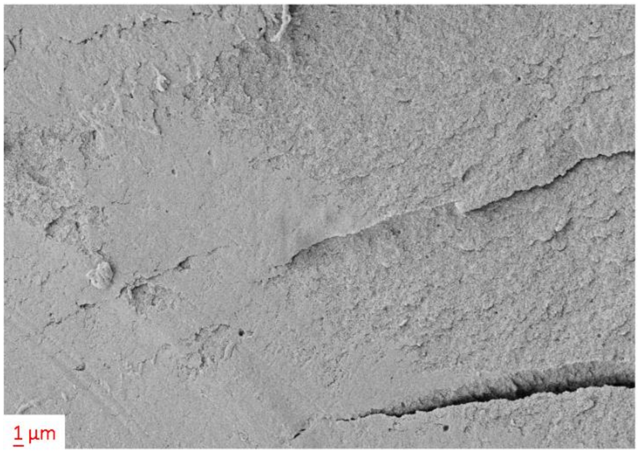

Figure S17. SEM picture of coating layer and area with open pores on aerogel surface after plasma treatment. WPI aerogel, coating: C_4F_8, mode: CW, p_{input} = 90 W, t = 50 min.

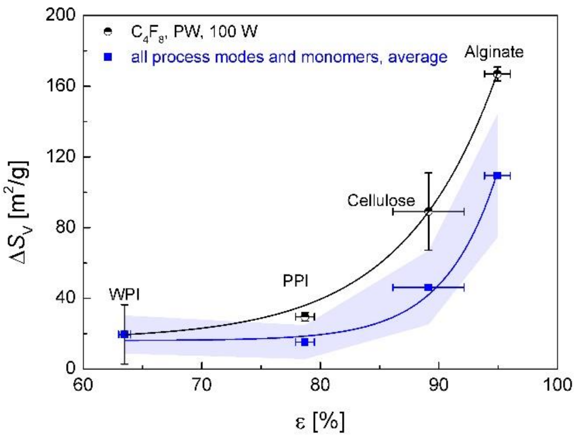

Figure S18. Increase of ΔSV in dependence of the overall porosity of the aerogels. Circles represent the values obtained with C_4F_8 monomer, in PW mode with p_{input} = 300 W. Squares represent the averaged values from all coating materials and process conditions, excluding PFAC-8 coating in CW mode with p_{input} = 90 W. Straight lines represent exponential fitting. Error areas represent the standard deviation of ΔSV from averaged values at different process conditions; error bars represent the relative measurement errors (x-error) and standard deviation of ΔSV for the individual process condition (y-error).

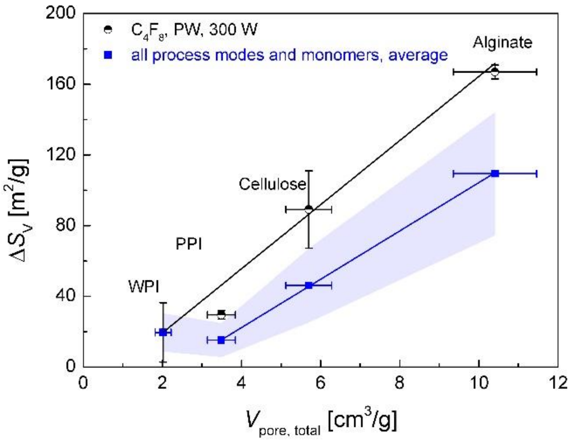

Figure S19. Increase of ∆SV in dependence of the overall pore volume of the aerogels. Circles represent the values obtained with C_4F_8 monomer, in PW mode with p_{input} = 300 W. Squares represent the averaged values from all coating materials and process conditions, excluding PFAC-8 coating in CW mode with p_{input} = 90 W. Straight lines represent exponential fitting. Error areas represent the standard deviation of ∆*S*V from averaged values at different process conditions; error bars represent the relative measurement errors (x-error) and standard deviation of ∆*S*V for the individual process condition (y-error).

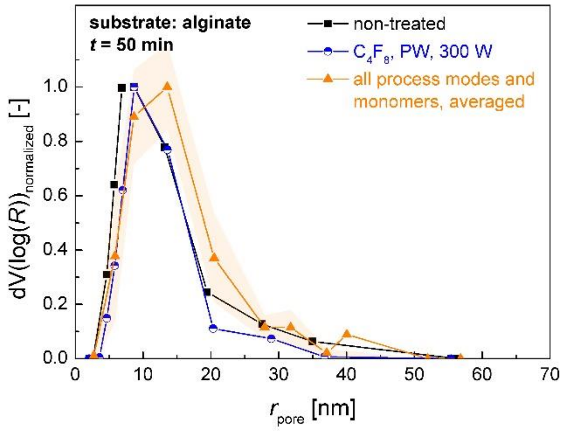

Figure S20. Comparison of BJH pore size distributions (normalized) from alginate aerogels prior and after the plasma coating process. Round data points represent the values obtained after coating with C_4F_8 in PW mode with p_{input} = 300 W, triangular data points represent the averaged values obtained after all other coating process conditions, excluding PFAC-8 coating in CW mode with pinput = 90 W. Error areas represent the standard deviation from averaged values.

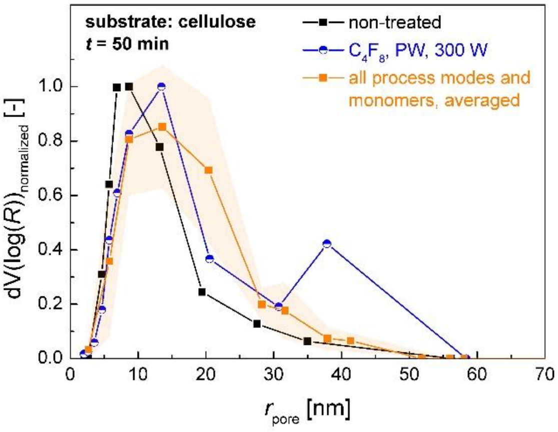

Figure S21. Comparison of BJH pore size distributions (normalized) from cellulose aerogels prior and after the plasma coating process. Round data points represent the values obtained after coating with C_4F_8 in PW mode with p_{input} = 300 W, triangular data points represent the averaged values obtained after all other coating process conditions, excluding PFAC-8 coating in CW mode with pinput = 90 W. Error areas represent the standard deviation from averaged values.

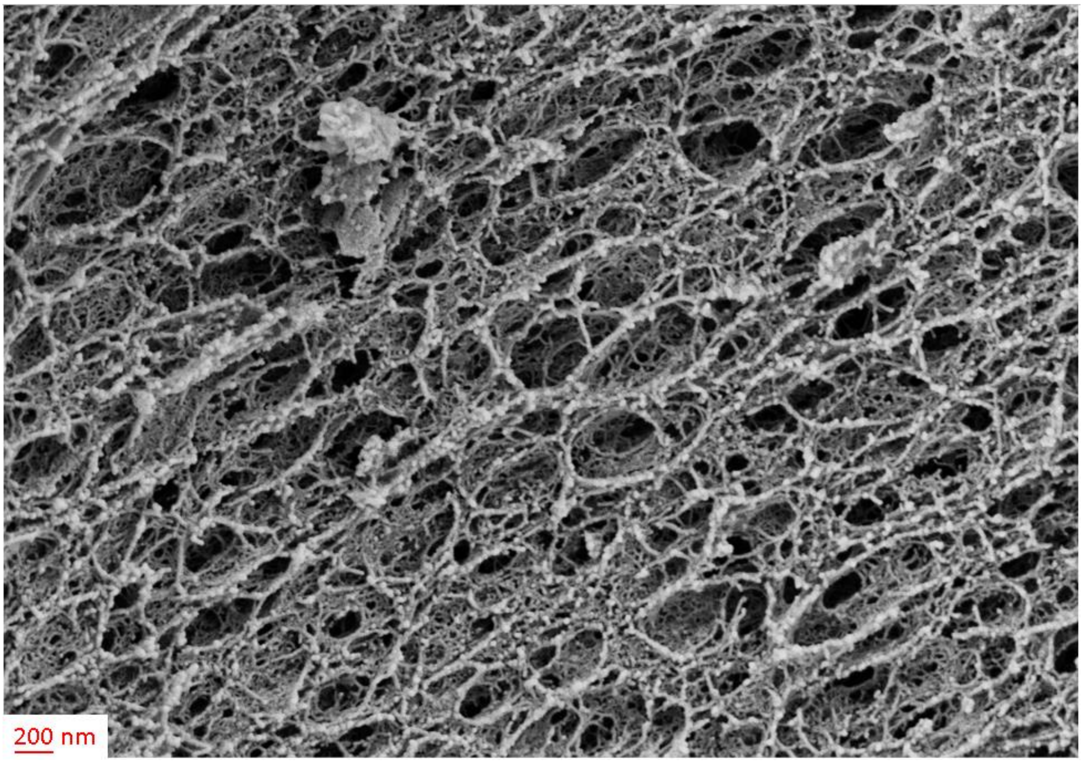

Figure S22. SEM picture of pore structure in alginate aerogel after plasma treatment. Coating: C_4F_8, mode: PW, p_{input} = 300 W, t = 20 min.

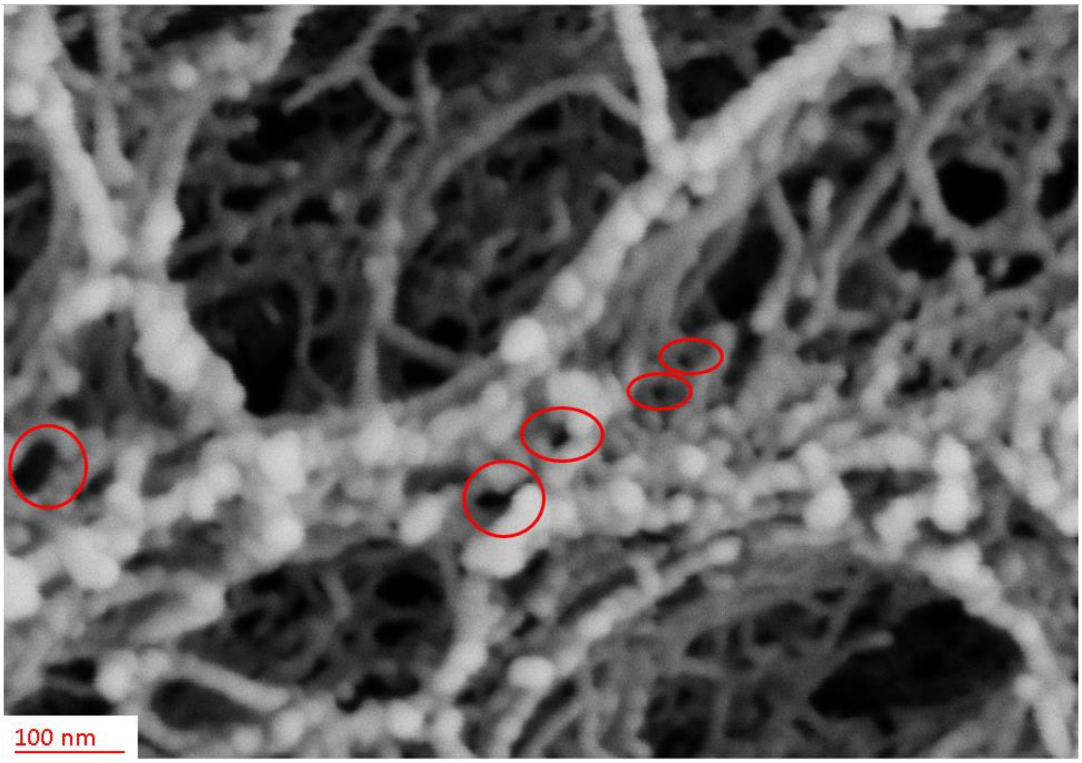

Figure S23. SEM picture of pore structure in alginate aerogel after plasma treatment. Coating: C_4F_8, mode: PW, p_{input} = 300 W, t = 20 min. Some mesopores formed from coating material are highlighted (red circles).

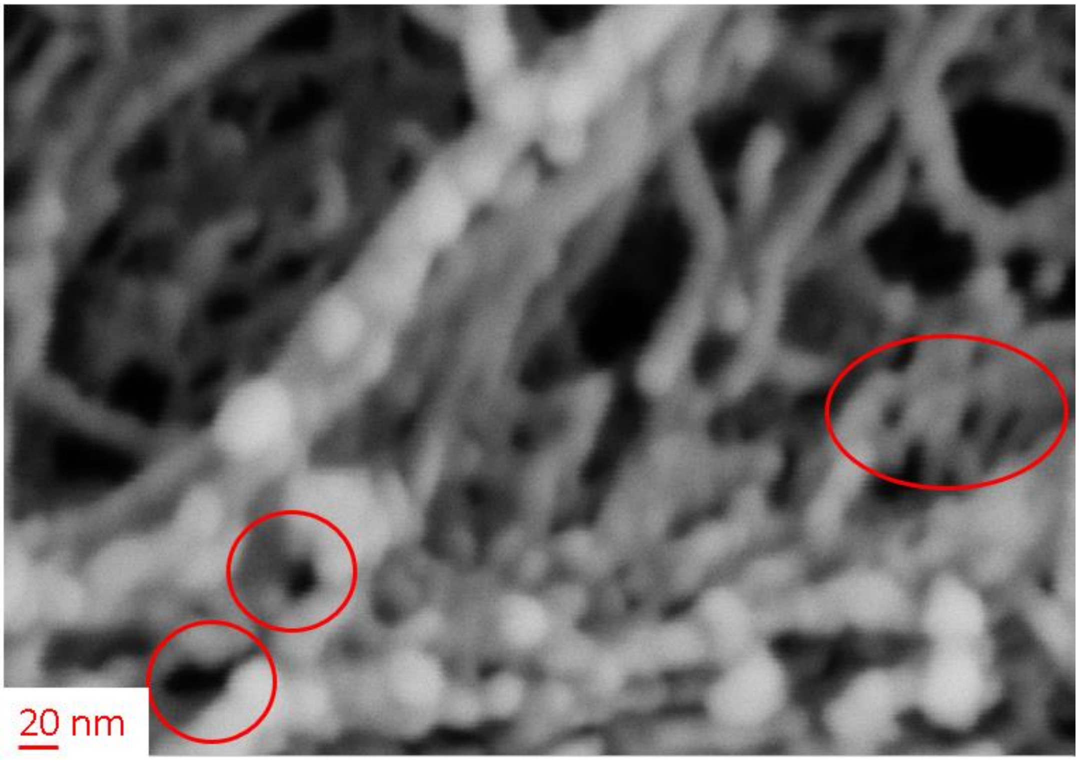

Figure S24. SEM picture of pore structure in alginate aerogel after plasma treatment. Coating: C_4F_8, mode: PW, p_{input} = 300 W, t = 20 min. Some mesopores formed from coating material are highlighted (red circles).
